# A powerful microbiome-based association test and a microbial taxa discovery framework for comprehensive association mapping

**DOI:** 10.1186/s40168-017-0262-x

**Published:** 2017-04-24

**Authors:** Hyunwook Koh, Martin J. Blaser, Huilin Li

**Affiliations:** 1 Department of Population Health and Environmental Medicine, New York University School of Medicine, New York, NY 10016 USA; 2 Department of Medicine and Microbiology, New York University Langone Medical Center, New York, NY 10010 USA

**Keywords:** Microbial association test, Microbial group analysis, Upper-level taxa, Taxonomic structure, Phylogenetic tree, Comprehensive association mapping

## Abstract

**Background:**

The role of the microbiota in human health and disease has been increasingly studied, gathering momentum through the use of high-throughput technologies. Further identification of the roles of specific microbes is necessary to better understand the mechanisms involved in diseases related to microbiome perturbations.

**Methods:**

Here, we introduce a new microbiome-based group association testing method, optimal microbiome-based association test (OMiAT). OMiAT is a data-driven testing method which takes an optimal test throughout different tests from the sum of powered score tests (SPU) and microbiome regression-based kernel association test (MiRKAT). We illustrate that OMiAT efficiently discovers significant association signals arising from varying microbial abundances and different relative contributions from microbial abundance and phylogenetic information. We also propose a way to apply it to fine-mapping of diverse upper-level taxa at different taxonomic ranks (e.g., phylum, class, order, family, and genus), as well as the entire microbial community, within a newly introduced microbial taxa discovery framework, microbiome comprehensive association mapping (MiCAM).

**Results:**

Our extensive simulations demonstrate that OMiAT is highly robust and powerful compared with other existing methods, while correctly controlling type I error rates. Our real data analyses also confirm that MiCAM is especially efficient for the assessment of upper-level taxa by integrating OMiAT as a group analytic method.

**Conclusions:**

OMiAT is attractive in practice due to the high complexity of microbiome data and the unknown true nature of the state. MiCAM also provides a hierarchical association map for numerous microbial taxa and can also be used as a guideline for further investigation on the roles of discovered taxa in human health and disease.

**Electronic supplementary material:**

The online version of this article (doi:10.1186/s40168-017-0262-x) contains supplementary material, which is available to authorized users.

## Background

The human microbiota is the set of all microorganisms inhabiting the human body [1]. Recently, their roles in human health and disease have been highlighted [2–9]. Advancement in studies of the microbiota has gathered momentum due to the advent of high-throughput sequencing technologies which enable microbiota profiling [10–12]. Since raw sequences preprocessed by these platforms include highly variable regions to be used as unique markers for each microbe, diverse microbes can be identified based on the sequence similarity and then assigned to operational taxonomic units (OTUs) [13]. These OTUs are characterized by their quantity, read count, or relative abundance, and the difference in microbial abundances may be associated with health or disease status [14, 15]. The phylogenetic tree illustrates taxonomical and evolutionary relationships among diverse microbes [13, 16, 17], and its related microbial complexity provides further insights about possible health and disease etiologies [18, 19]. Further identification of microbial taxa is needed to better understand the relationship of particular microbiota with human health and disease. It is also common that recent studies report discovered upper-level taxa at a high taxonomic rank (e.g., phylum, class, order, family, and genus) along with the dynamics of the entire microbial community complexity instead of enumerating individual microorganisms. The upper-level taxa can be considered as groups of various nested lineages. Hence, likewise the entire microbial community, numerous statistical challenges can arise to analyze them properly [20]. Nevertheless, a conventional ecological method, referenced as the aggregate-based method in this paper, is most commonly used for association testing [8, 9, 21–24]. The aggregate-based method is based on a univariate analysis, using aggregates of microbial abundances in a lower-level lineage per sample as a single predictor variable. It is also regarded as an approach equipped with the popular methods, the linear discriminant analysis effect size (LEfSe) [21], STAMP [22], DESeq2 [23], and metagenomeSeq-fit Zig [24]. The major problem of this approach is its underlying assumption that associated OTUs nested at each upper-level taxon are all in the same effect direction. Any violation of this assumption can lead to a substantial loss of power.

As a counterpart to the aggregate-based method, we investigate two existing methods, microbiome regression-based kernel association test (MiRKAT) [25] and microbiome-based sum of powered score tests (MiSPU) [26], and propose a new method, optimal microbiome-based association test (OMiAT), for more sophisticate microbial association testing. Recently, MiRKAT has been spotlighted in the literature because of its comprehensive capability to incorporate diverse distance-based measures, including the unique fraction (UniFrac) distance [27–29] and the Bray-Curtis dissimilarity, into its kernel machine regression framework [30]. The distance-based measures integrate different relative contributions from microbial abundance and phylogenetic tree information, and thus, they suit different association patterns, respectively [25, 27–29]. In practice, any strong biological evidence inclined to a particular distance-based measure is usually absent; hence, the data-driven approach of MiRKAT, Optimal MiRKAT, which uses an optimal test among different distance-based measure trials, is highly attractive. On the other hand, MiSPU is constructed on the sum of powered score tests (SPU) framework [31] based on a newly defined measure, generalized taxon proportion [26]. Similar to unweighted and weighted UniFrac distances [27, 28], Wu et al. [26] describe that two different versions of the generalized taxon proportion, unweighted and weighted generalized taxon proportion, are suitable for discovering rare and common/abundant taxa, respectively. Wu et al. [26] also insist that the data-driven approach of MiSPU, adaptive MiSPU (aMiSPU), is robust and powerful by taking a highly adaptive test, utilizing the variable selection/weighting of the SPU framework based on the two generalized taxon proportions, comprehensively. However, we argue that the unweighted generalized proportion might not be sufficient to account for varying microbial abundances because it is based on the presence or absence of microbial taxa with no further microbial abundance information incorporation. In addition, since the generalized taxon proportion weights microbial taxa by their branch lengths [26], its weighting scheme might be efficient only when associated microbial taxa have relatively large branch lengths.

Our proposed method, OMiAT, is a data-driven testing method which takes an optimal test through diverse tests from both SPU and MiRKAT. To avoid confusion, we explain here that the SPU used for OMiAT is different from MiSPU in that it is not based on the generalized taxon proportion but implemented on standardized compositional data with no phylogenetic information incorporation. We have first been convinced that MiRKAT is suitable to modulate relative contributions from microbial abundance and phylogenetic information by the use of diverse distance-based measures. However, we emphasize that SPU is advantageous over MiRKAT to modulate different association patterns arising from highly imbalanced microbial abundances, utilizing its wide range of power value choices [31]. Consequently, OMiAT is highly efficient to discover significant association signals from diverse underlying association patterns and thus attractive in practice due to the high complexity of microbiome data and the unknown true nature of the state. Our extensive simulations and real data analyses also demonstrate more robust and powerful performance of OMiAT, compared with other competing methods.

We also introduce a microbial taxa discovery framework, namely, microbiome comprehensive association mapping (MiCAM), which uses different configurations to fine-map diverse microbial taxa throughout all taxonomic ranks, comprehensively. MiCAM tests all upper- and lower-level taxa and applies multiple testing correction per taxonomic rank. MiCAM incorporates OMiAT as a group analytic method for assessing upper-level taxa. MiCAM discovers significantly associated taxa and controls false discovery rate at 5% per taxonomic rank. Testing numerous microbial taxa individually may lead to a huge computational burden. Thus, we apply a combined permutation-based algorithm to MiCAM to obtain stable outcomes (e.g., *P* values) in a computationally manageable manner. A newly introduced visualization approach for MiCAM also helps to organize discovered microbial taxa hierarchically.

The methodological aspects of OMiAT and MiCAM can be found in the following “Methods” section. Extensive simulation experiments and real data analyses are addressed in the “Results” section.

## Methods

### Models and notations

This section is devoted to describe the methodological aspects of OMiAT and how its performance is affected in microbial group analysis. Since OMiAT is based on two existing methods, SPU [31] and MiRKAT [25], we start with the descriptions of SPU and MiRKAT. OMiAT shares some of the useful features of SPU and MiRKAT, as follows. OMiAT is based on a generalized linear model framework so that different types of outcome traits, such as continuous and binary responses, with potential covariate adjustments, can be handled. OMiAT is also based on score tests which do not require any statistical estimation for the parameters of major interest [32].

Suppose the data include *n* subjects, *p* OTUs, and *q* covariates (e.g., environmental factors) and the subscripts, *i*, *j*, and *k*, indicate a subject, an OTU, and a covariate, respectively. Then, an *n* × 1 vector, Y, for the outcome response, either as a form of continuous or binary traits, is marked asY_*i*_ for *i* = 1, …, *n*, an *n* × *p* matrix, Z, for the OTUs in a microbial group is marked as Z_*ij*_ for *i* = 1,…, *n* and *j* = 1,…, *p*, and an *n* × *k* matrix, X, for the covariates is marked as X_*ik*_ for *i* = 1,…, *n* and *k* = 1, …, *q*.

To relate OTUs with an outcome response while adjusting for covariates, we consider a multiple regression model (Eq. 1) for continuous traits and a multiple logistic regression model (Eq. 2) for binary traits.

1 $$ {\mathrm{Y}}_i={\upbeta}_0+{\displaystyle {\sum}_{k=1}^q}{{\mathrm{X}}_i}_k{\alpha}_k+{\displaystyle {\sum}_{j=1}^p}{\mathrm{Z}}_{i j}{\upbeta}_j+{\in}_i, $$

where ∈_i_ is an error term which is independently and identically distributed with a mean zero and a variance of σ^2^.

2 $$ \mathrm{logit}\kern0.5em  P\left({\mathrm{Y}}_i=1\right)={\upbeta}_0+{\displaystyle {\sum}_{k=1}^q}{{\mathrm{X}}_i}_k{\upalpha}_k+{\displaystyle {\sum}_{j=1}^p}{{\mathrm{Z}}_i}_j{\upbeta}_{\mathrm{j}}. $$

Then, the score vector to test the null hypothesis of no association between OTUs and an outcome trait, H_0_ : β = (β_1_, …, β_*p*_) ' = 0,_,_ is given as in Eq. 3.

3 $$ \mathrm{U}=\left({\mathrm{U}}_1,\dots, {\mathrm{U}}_p\right)\hbox{'}=\left({\displaystyle {\sum}_{i=1}^n}\left({\mathrm{Y}}_i\hbox{-} {\widehat{\upmu}}_{i,0}\right){{\mathrm{Z}}_i}_1,\dots, {\displaystyle {\sum}_{i=1}^n}\left({\mathrm{Y}}_i\hbox{-} {\widehat{\upmu}}_{i,0}\right){{\mathrm{Z}}_i}_p\right)\hbox{'}, $$

where ${\widehat{\upmu}}_{i,\ 0}$ is the predicted value of Y_i_ under H_0_ which can be estimated as ${\widehat{\upbeta}}_0+{\displaystyle {\sum}_{k=1}^q}{{\mathrm{X}}_i}_k{\widehat{\upalpha}}_k$ for continuous traits or logit^−1^ $\left({\widehat{\upbeta}}_0+{\displaystyle {\sum}_{k=1}^{\mathrm{q}}}{{\mathrm{X}}_i}_k{\widehat{\upalpha}}_k\right)$ for binary traits, where ${\widehat{\upbeta}}_0$ and ${\widehat{\upalpha}}_k$ are maximum likelihood estimates under H_0_ [33–35].

#### SPU [31]

Pan et al. [31] formulated their method, SPU, with its test statistic as in Eq. 4 to obtain a generalized framework to sum individual score components to be powered with diverse *γ* value choices (*γ* ≥ 1, integer).

4 $$ {\mathrm{T}}_{\mathrm{SPU}\left(\upgamma \right)}={\displaystyle {\sum}_{j=1}^p}{\mathrm{U}}_j^{\gamma} $$

SPU was originally proposed for gene- or region-based association testing in genome-wide association studies. SPU in our proposed method, OMiAT, is implemented on the standardized compositional data (i.e., starting from the form of compositional data (i.e., percentages), for each OTU, we subtract its mean from individual raw percentages and then divide the difference by its standard deviation) because of varying total reads per sample.

In microbial group analysis, the SPU test using an odd value of *γ* is suitable when associated OTUs have the same effect direction, while the SPU test using an even value of *γ* is more suitable when those are in mixed effect directions [31, 33, 36]. To explain, when *γ* is an odd number, the score components in the SPU test can be canceled out in its final summing stage by the existence of opposite directional components, which results in a significant loss of power in the mixed effect situations. Instead, when *γ* is an even number, those in the SPU test can be protected, which result in a powerful performance in the mixed effect directions. As *γ* increases, relatively larger score components will gradually be weighted more, while relatively small score components will gradually be ignored [31]. We can see that the score statistic value is affected by OTU abundance as in Eq. 3. Thus, a situation in which abundant OTUs are associated indicates that their corresponding components in the score vector are large, and thus, SPU using a large value of *γ* is more suitable by weighting them more and removing noisy small signals from the others. In contrast, when associated OTUs are rare in abundance, indicating small score components, SPU using a small value of *γ* can be more suitable by preserving them in the final aggregate. Since, in most microbiomes, OTU abundance is highly imbalanced across individual OTUs, the high receptivity of SPU to a wide range of *γ* value choices must be maximized in microbial group analyses.

Therefore, the performance of SPU differs according to *γ* and the true underlying association patterns. Since we cannot predict which situation is related to our study in advance, the adaptive SPU (aSPU) test (Eq. 5) which takes the minimum *P* value among different *γ* value trials can be importantly considered [31].

5 $$ {\mathrm{T}}_{\mathrm{aSPU}}={min}_{\upgamma \upepsilon \Gamma}{\mathrm{P}}_{\mathrm{SPU}\left(\upgamma \right)} $$

The *γ* value can take any natural number (i.e., Γ = ℕ), but we used the candidate set, Γ = {1,2,3,4,5,6,7,8, ∞}, where T_SPU(∞)_ = max{|U_*j*_|, *j* = 1,......, *p*) [31], in our simulations and real data analyses and it was sufficient.

#### MiRKAT [25]

MiRKAT has recently been introduced to the microbiome research community for microbial community-level association testing. Here, we describe its key formula and ideas and refer to the original paper [25] for more details. MiRKAT is built on the kernel machine regression framework with the kernel formula, Eq. 6 [25, 30], to incorporate diverse distance-based measures, such as UniFrac distances [27–29] and Bray-Curtis dissimilarity.

6 $$ \mathrm{K}=\hbox{-} \frac{1}{2}\left( I\hbox{-} \frac{1{1}^{\prime }}{n}\right){D}^2\left( I\hbox{-} \frac{1{1}^{\prime }}{n}\right), $$

where *D* is the *n* × *n* pairwise distance matrix and *D* ^2^ is its element-wise square, *I* is the *n* × *n* identity matrix, and 1 in 11′ is the column vector of *n* ones. We can specify the pairwise distance matrix, *D*, in this kernel formula, choosing among diverse distance-based measures. Then, using the resulting kernel, the variance component score statistic can be formulated with Eq. 7 below.

7 $$ {\mathrm{Q}}_{\mathrm{MiRKAT}(k)} = \frac{1}{2\Phi}\ \left(\mathrm{Y} - {\widehat{\upmu}}_0\right)\hbox{'}{\mathrm{K}}_{(k)}\left(\mathrm{Y} - {\widehat{\upmu}}_0\right), $$

where Φ is the dispersion parameter which can be estimated as $\Phi ={\widehat{\upsigma}}_0^2$, where ${\widehat{\upsigma}}_0^2$ is the estimated residual variance under H_0_, for continuous traits, and as Φ = 1 for binary traits; Y − ${\widehat{\upmu}}_0$ is the vector, $\left({\mathrm{Y}}_1-{\widehat{\upmu}}_{1,0},\dots, {\mathrm{Y}}_n-{\widehat{\upmu}}_{n,0}\right)$; and *k* is an index for a particular kernel based on a chosen distance-based measure.

Of importance is that different distance-based measures suit different association patterns, respectively [25, 27–29]. The UniFrac distances are constructed on the basis of phylogenetic tree information and modulate the extent of microbial abundance to be incorporated by different weighting schemes [27–29]. Thus, the UniFrac distances are suitable when associated OTUs are phylogenetically related. Then, the unweighted UniFrac distance [27] is suitable for considering rare lineages, while the weighted UniFrac distance [28] can be used for studying common/abundant lineages. The generalized UniFrac distance [29] is a compromise version; thus, its use can also be modulated according to its parameter value. When associated OTUs are not phylogenetically related, the Bray-Curtis dissimilarity can be best because it is constructed based solely on microbial abundance not incorporating phylogenetic tree information. In terms of relative contribution from microbial abundance and phylogenetic tree information, we can also understand that the Bray-Curtis dissimilarity is most inclined to microbial abundance, and then, the weighted, generalized, and unweighted UniFrac distances follow in the name ordered. In practice, prior knowledge about the true underlying association patterns of numerous OTUs is likely to be absent; thus, Zhao et al. [25] have proposed the data-driven approach of MiRKAT, Optimal MiRKAT, which takes the minimum *P* value among multiple distance-based measure trials as in Eq. 8.

8 $$ {\mathrm{Q}}_{\mathrm{OMiRKAT}}={min}_{k\upepsilon \left\{1, \dots,\ \mathrm{l}\right\}}{\mathrm{P}}_{\mathrm{MiRKAT}(k)} $$

where P_MiRKAT(k)_ is the *P* value of the MiRKAT test based on Q_MiRKAT(k)_. We used seven candidate distance-based measures, Bray-Curtis dissimilarity, unweighted UniFrac, weighted UniFrac, four different generalized UniFrac measures with parameter values, 0, 0.25, 0.5, and 0.75, respectively, in our simulations and real data analyses.

#### OMiAT

OMiAT takes the minimum *P* value from all the score tests for SPU (i.e., T_SPU(γ)_ in Eq. 4) and MiRKAT (i.e., Q_MiRKAT(k)_ in Eq. 7) as its test statistic and can be simply expressed in Eq. 9.

9 $$ {\mathrm{M}}_{\mathrm{OMiAT}}= m i n\left\{{\mathrm{T}}_{\mathrm{aSPU}},{\mathrm{Q}}_{\mathrm{OMiRKAT}}\right\} $$

Consequently, OMiAT is highly robust and powerful by taking an optimal test from all different tests for varying microbial abundances by SPU and for different relative contributions from microbial abundance and phylogenetic information by MiRKAT. Of course, we do not use the genuine minimum *P* value, M_OMiAT_, to be reported as a final outcome *P* value, but it is a test statistic to be used for estimating a *P* value. As with Pan et al. [31] and Zhao et al. [25], we also use a permutation-based method [37] to calculate *P* values for the test statistics, T_SPU(γ)_, T_aSPU_, Q_MiRKAT(k)_, Q_OMiRKAT_, and M_OMiAT_. Detailed information on it is addressed in Additional file 1: Table S1.

### Aggregate-based method

There exist different aggregate-based methods, LEfSe [21], STAMP [22], DESeq2 [23], and metagenomeSeq-fit Zig [24]. LEfSe and STAMP employ the non-parametric Kruskal-Wallis test [21, 22, 38] as a univariate analytic method. This non-parametric method is designed for one-way layout data structure, and thus, it is difficult to handle covariate adjustments (e.g., environmental factors). Moreover, this method cannot analyze continuous outcome traits; hence, its usability is limited. For DESeq2 and metagenomeSeq-fit Zig, the main assumption of their parametric methods may not be validated due to the issue of relative abundance, which can result in inflated type I error rates [16, 39]. For these reasons, we do not consider these existing machineries, as they are, for the aggregate-based method. Instead, we employ a semi-parametric approach which is based on a score test and a permutation-based method for the aggregate-based method to be investigated in our simulations and real data analyses. It begins with the standardized compositional data and aggregates it per sample. Then, using a resulting single predictor variable for the aggregates and an outcome variable, we estimate *P* values based on the score test statistic, *U*, in Eq. 3 and a permutation-based method [37].

### MiCAM

In this section, we illustrate a new microbial taxa discovery framework, MiCAM, to fine-map diverse microbial taxa from the highest (e.g., kingdom/the entire community) to the lowest (e.g., species) taxonomic rank. MiCAM tests all microbial taxa using different configurations for the assessment of upper-level taxa, taxa in the species taxonomic rank, and taxa that include only one OTU, respectively, and applies multiple testing correction per taxonomic rank. We also describe its testing algorithm and graphical representation.

#### Assessment of upper-level taxa

The upper-level taxa at different taxonomic ranks (e.g., kingdom, phylum, class, order, family, and genus) are a group of individual OTUs except for a few which include only one OTU. Thus, we apply OMiAT to assess the upper-level taxa by sub-grouping OTUs and pruning a phylogenetic tree for the ones nested in each of the upper-level taxa. For small upper-level taxa, including only a few OTUs, the UniFrac distances may not be computed when there is no phylogenetic disparity for any pairwise sample comparison. For this case, the Optimal MiRKAT part in Eq. 9 is replaced with the MiRKAT based on a single kernel for the Bray-Curtis dissimilarity.

#### Assessment of taxa in the species taxonomic rank

The species taxonomic rank may not be regarded as a microbial group but as individual microbes. However, we include this species rank to be analyzed because testing separately for individual species is also of interest. Although it might be considered ideal to have one-to-one correspondence between OTUs and species, in reality, some species include multiple OTU. As such, we can consider an OTU as the smallest unit and thus those species as a group so that OMiAT is applied. Alternatively, users can consider any species as the smallest unit, by properly combining the relevant OTUs per species.

#### Assessment of taxa that include only one OTU

Group analytic tools lose their efficiency when the taxa surveyed include only one OTU. For this case, we employ a simple semi-parametric approach which is based on a score test, *U* (Eq. 3), and a permutation-based method [37].

#### Control of false discovery rate

Importantly, since there can exist multiple tests for multiple taxa at a given taxonomic rank, a multiple testing correction procedure is needed to suppress increased type I error rate. We apply the Benjamini-Hochberg procedure to control false discovery rate at 5% per taxonomic rank [40–42] as it is valid robustly whenever the multiple tests are independent or correlated in various scenarios [43]. Thus, the error probability applies to a family of inferences at each taxonomic rank.

#### A combined permutation-based algorithm

One issue we have encountered is a large computational burden to test numerous microbial taxa throughout all different taxonomic ranks. Although the score-based test using a permutation-based method is efficient in computation to test a small number of microbial taxa, testing all existing microbial taxa can require enormous computational time. Moreover, to reach sufficient convergence in all the outcomes (e.g., *P* values) consistently, computational needs can be even greater.

Our experiences have also revealed that outcomes are sensitive to different implementation specifications (e.g., different numbers of permutations). Accordingly, especially when the *P* values are close to 0.05, their discovery status can even be reversed. Therefore, we apply a combined permutation-based algorithm which shares the same vector and permuted vectors of residuals for every microbial taxon assessment; hence, we can avoid repeating such procedures. We have found that this combined permutation-based algorithm produces stable outcomes (e.g., *P* values) with better correspondence/convergence than individual permutation-based tests for numerous assessments using a relatively moderate number of permutations (e.g., 50,000). In contrast, individual permutation-based tests may produce highly irregular outcomes unless an extremely high number of permutations (e.g., 500,000) are specified.

#### A hierarchical visualization

A graphical representation is introduced to summarize discovered and undiscovered microbial taxa in a hierarchical taxonomic structure. To explain, we stack all individual OTUs vertically and enumerate taxonomic ranks from highest to lowest horizontally with each OTU consistently belonging to their upper-level taxa. Then, using color, we highlight microbial taxa according to their discovery status to overview multiple discovery statuses comprehensively. In addition, on the right end line on each graph, we enumerate the effect directions for each OTU by calculating the score test statistic, *U* (Eq. 3), for each OTU, assigning “+”, if it is ≥0 and “−” if it is <0. Related outcomes are presented in the “Real data analysis” section. Detailed information on the exact taxonomic names and their *P* values matched with each OTU ID can also be found in separate tables using our software facility, OMiAT.

#### Other methods in MiCAM

Although we propose OMiAT to be used as a group analytic method in MiCAM, for the purpose of comparison in our real data analyses, we have also integrated other competing group analytic methods, Optimal MiRKAT, aMiSPU, and the aggregate-based method, respectively, into the MiCAM framework.

## Results

### Simulations

We have conducted extensive simulations to evaluate different methods, OMiAT, Optimal MiRKAT, aMiSPU, and the aggregate-based method in terms of type I error and statistical power. For simplicity, we use the entire community as a microbial group of interest. In practice, any subgroup for different upper-level taxa can be considered.

#### Simulation design

The simulation design used is based on the prior studies [24, 25, 30]. We first simulated OTU counts for 100 subjects from the Dirichlet-multinomial distribution with total reads per sample to be randomly sampled from a negative binomial distribution with mean 300 and size 10. The dispersion parameter and proportion means to be inserted into the Dirichlet-multinomial distribution were estimated from the early childhood antibiotics and the microbiome (ECAM) project’s intestinal microbiome data [7]. The ECAM data includes 2261 OTUs for 43 infants, but as a demonstration, we selected 32 infants aged from 30 to 40 days of life and applied a filtering rule that retains only OTUs with a proportion mean >10^−3^, as such, 71 OTUs were included in the analysis. Then, continuous and binary outcome traits were generated under the linear model (Eq. 10) and the logistic model (Eq. 11), respectively.

10 $$ {\mathrm{y}}_i=0.5*\mathrm{scale}\left({\mathrm{X}}_{1 i}+{\mathrm{X}}_{2 i}\right)+{\displaystyle {\sum}_{j=1}^p{\upbeta}_j\mathrm{scale}\left({\mathrm{Z}}_{i j}\right)}+{\in}_i $$

11 $$ \mathrm{logit}\ \mathrm{P}\left({\mathrm{y}}_i=1\right)=0.5*\mathrm{scale}\left({\mathrm{X}}_{1 i}+{\mathrm{X}}_{2 i}\right)+{\displaystyle {\sum}_{j=1}^p}{\upbeta}_j\mathrm{scale}\left({\mathrm{Z}}_{i j}\right), $$

where ϵ_i_ is an error term with ϵ_i_ ~ N(0,1), X_1i_ and X_2i_ are two covariates, Z_ij_ is an OTU count, and the “scale” function is for the standardization to have mean 0 and standard deviation (SD) 1 and is further defined as scale $\left({\mathrm{X}}_{1\mathrm{i}}+{\mathrm{X}}_{2 i}\right) = \frac{{\mathrm{X}}_{1 i} + {\mathrm{X}}_{2 i} - \mathrm{mean}\left({\mathrm{X}}_{11} + {\mathrm{X}}_{21},\kern0.5em {\mathrm{X}}_{12} + {\mathrm{X}}_{22}, \dots,\ {\mathrm{X}}_{1 n} + {\mathrm{X}}_{2 n}\right)}{\mathrm{SD}\left({\mathrm{X}}_{11} + {\mathrm{X}}_{21},\kern0.5em {\mathrm{X}}_{12} + {\mathrm{X}}_{22}, \dots,\ {\mathrm{X}}_{1 n} + {\mathrm{X}}_{2 n}\right)}$ and $\mathrm{scale}\left({\mathrm{Z}}_{ij}\right) = \frac{{\mathrm{Z}}_{ij} - \mathrm{mean}\left({\mathrm{Z}}_{1 j},\ {\mathrm{Z}}_{2 j}, \dots,\ {\mathrm{Z}}_{nj}\right)}{\mathrm{SD}\left({\mathrm{Z}}_{1 j},\ {\mathrm{Z}}_{2 j}, \dots,\ {\mathrm{Z}}_{nj}\right)},$ for subjects *i* = 1,…, *n* and OTUs j = 1,…, *p*. X_1*i*_ ' s were generated to be independent with OTUs from the Bernoulli distribution with success probability 0.5. X_2*i*_ ' s were generated in two different ways: one to be correlated with OTUs as X_2*i*_ = ∑_*j* ∈ Λ_scale(Z_*ij*_) + N(0, 1), where Λ is a set of indices for associated OTUs, and the other to be independent with OTUs as X_2i_ = N(0,1).

To estimate type I error rates, outcome traits were generated from the null model by setting β = (β_1_, …, β_*p*_) ' = 0. To estimate statistical powers, we first selected a set of associated OTUs with four different simulation scenarios: (1) OTUs in upper 10% in abundance, (2) a random 10% of OTUs, (3) OTUs in lower 10% in abundance, and (4) OTUs in the selected cluster using the partitioning-around-medoids (PAM) algorithm [44]. The fourth scenario is for a situation when associated OTUs are phylogenetically related. For this, we first partitioned all OTUs into five clusters using the PAM algorithm based on the cophenetic distances in the real phylogenetic tree [45]. Then, we randomly assigned all these five clusters (which contain 20.9, 21.6, 32.5, 15.3, and 9.7% of total abundance, respectively) into each iteration in our simulations. This is to overcome the arbitrariness of the choice of clusters and opposite to working on a single or a couple of chosen cluster(s) as conducted in prior studies [24, 26, 30]. If we work on simulations with some particularly chosen clusters, it would not be a fair comparison because those clusters can be favorable to a particular testing method. Especially, adaptive methods are needed to be tested from diverse simulation environments (e.g., differing microbial abundances and phylogenetic relationships using different associated clusters evenly) to evaluate their adaptivity.

Because the fourth scenario combines both microbial abundance and phylogenetic information, it is believed to be more realistic than the first three scenarios. However, the first three scenarios are useful to check whether each method discovers abundant or rare microbial taxa, equivalently. We denote Λ as a set of indices for the associated OTUs. Then, β_*j* ∈ Λ_ is a vector of coefficients corresponding to associated OTUs. For each experimental setting, β_*j* ∈ Λ_ are simulated with three different continuous uniform distributions, Uniform(0,1), Uniform(0,2), and Uniform(0,3), for the same effect direction and with another three continuous uniform distributions, Uniform(−1,1), Uniform(−2,2), and Uniform(−3,3), for mixed effect directions, separately.

#### Simulation results

For presentation, we include only the outcomes for the adaptive methods (with the exception of the aggregate-based method) and for the logistic models, moving all the other outcomes to additional material (Additional file 2: Figure S1 reports complete type I error estimates, Additional files 3 and 4: Figure S2 and S3 report complete power estimates for the linear models, and Additional files 5 and 6: Figure S4 and S5 report complete power estimates for the logistic models).

##### Type I error

First, we observe mostly well-controlled type I error rates (≤~5%) across all methods [Table 1, Additional file 2: Figure S1]. Therefore, any discovered microbial taxa using any of these methods are from statistically valid approaches.

**Table 1:** Type I error rate estimates in percent for both linear and logistic models

		OMiAT	Optimal MiRKAT	aMiSPU	Aggregate-based
Linear model	Independent X_2_	4.98	5.10	5.13	4.84
	Correlated X_2_	4.93	4.92	5.15	4.92
Logistic model	Independent X_2_	5.09	4.98	5.09	5.10
	Correlated X_2_	5.26	5.21	4.94	4.99

##### Power

**Fig. 1:**
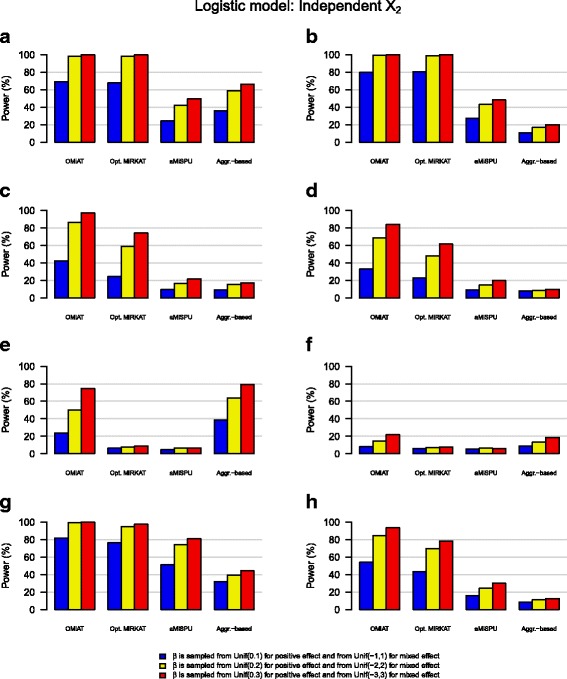
Power estimates for the logistic model when the covariate X_2_ is independent with OTUs. **a** Positive effect: OTUs in upper 10% in abundance. **b** Mixed effect: OTUs in upper 10% in abundance. **c** Positive effect: a random 10% of OTUs. **d** Mixed effect: a random 10% of OTUs. **e** Positive effect: OTUs in lower 10% of abundance. **f** Mixed effect: OTUs in lower 10% of abundance. **g** Positive effect: OTUs in the cluster. **h** Mixed effect: OTUs in the cluster

**Fig. 2:**
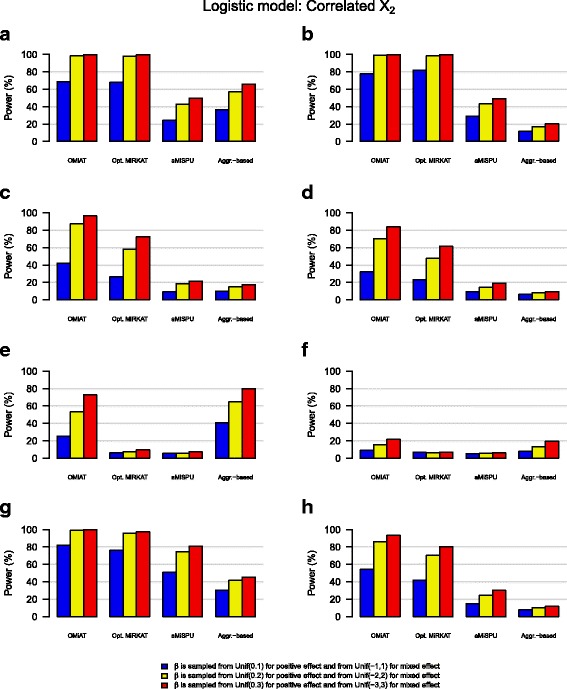
Power estimates for the logistic model when the covariate X_2_ is correlated with OTUs. **a** Positive effect: OTUs in upper 10% in abundance. **b** Mixed effect: OTUs in upper 10% in abundance. **c** Positive effect: a random 10% of OTUs. **d** Mixed effect: a random 10% of OTUs. **e** Positive effect: OTUs in lower 10% of abundance. **f** Mixed effect: OTUs in lower 10% of abundance. **g** Positive effect: OTUs in the cluster. **h** Mixed effect: OTUs in the cluster

We observe that with the increase of effect size, the power increases for all methods under any simulation scenario [Figs. 1 and 2, Additional files 3, 4, 5, and 6: Figure S2–S5]. We also observe that power is generally higher for linear than logistic models [Additional files 3, 4, 5, and 6: Figure S2–S5], but the relative performance among different methods within the linear or logistic model remains similar. Whether the covariate, X_2_, is independent or correlated with OTUs does not strongly alter the relative performance.

We observe that OMiAT is clearly more powerful than the other methods under most of the scenarios [Figs. 1 and 2]. Exceptions include situations where abundant OTUs are associated, in which Optimal MiRKAT is most powerful [Figs. 1 and 2a, b], and where rare OTUs are associated, in which the aggregate-based method is most powerful [Figs. 1 and 2e, f]. However, even then, OMiAT is highly comparable. Based on the first three scenarios, we can observe that SPU using a high *γ* value (≥4) is powerful when abundant OTUs are associated [Additional files 3, 4, 5, and 6: Figure S2–S5: A, B], SPU using a medium *γ* value (~4) is powerful when random OTUs are associated [Additional files 3, 4, 5, and 6: Figure S2–S5: C, D], and SPU using a low *γ* value (≤4) is powerful when rare OTUs are associated [Additional files 3, 4, 5, and 6: Figure S2–S5: E, F]. In contrast, the major drawback of Optimal MiRKAT occurs when rare or random OTUs are associated, resulting in low power values [Figs. 1c–f and 2c–f]. Consequently, we can observe that OMiAT reaches the highest power considerably beyond Optimal MiRKAT for the fourth scenario [Figs. 1g, h and 2g, h], as explained by the assistance from diverse SPU tests within its machinery.

The aggregate-based method is highly underpowered when associated OTUs are in mixed effect directions [Figs. 1b, d, f, h and 2b, d, f, h], as explained by the violation of its underlying assumption that all associated OTUs are in the same effect direction. Moreover, the aggregate-based method is less powerful than the other methods under most of the other scenarios [Figs. 1a, c, g and 2a, c, g]. The only exception is when rare OTUs are associated and they are in the same effect direction [Figs. 1e and 2e], which can be explained similarly with the situation where the SPU test using a small value of *γ* outperforms.

aMiSPU is not observed to be as powerful as Optimal MiRKAT as well as OMiAT [Figs. 1 and 2], and it is opposite to the simulation outcomes reported in Wu et al. [26]. For the reasons, we can further observe two related simulation outcomes as follows. Firstly, the MiSPU tests based on the unweighted generalized taxon proportion are mostly underpowered [Additional files 3, 4, 5, and 6: Figure S2–S5]. This may be because it is solely based on the presence or absence of microbial taxa with no further microbial abundance incorporation. Secondly, the MiSPU tests based on the weighted generalized taxon proportion are less powerful than the MiRKAT tests based on different UniFrac distances [Additional files 3, 4, 5, and 6: Figure S2–S5]. This may be because the generalized taxon proportion weights microbial taxa by their branch lengths [26], and thus, it is efficient only when associated microbial taxa have relatively large branch lengths, but not in general. In addition, in Wu et al. [26], a limited number of candidate distance-based measures were surveyed for different MiRKAT tests, which can lead to a lower power for Optimal MiRKAT [26].

### Real data analysis

Here, we apply the methods, OMiAT, Optimal MiRKAT, aMiSPU, and the aggregate-based method, respectively, to the MiCAM framework to assess existing microbial taxa throughout all different taxonomic ranks from kingdom to species using two real data sets [6, 7]. Along with the simulation results, we also compare different methods by the extent of discovered microbial taxa from our real data analyses.

#### Sustained effects on intestinal microbiota by early-life low-dose penicillin exposure [6]

Cox et al. [6] have conducted a microbiome profiling study to examine whether the intestinal microbiota altered during maturation by low-dose antibiotic, low-dose penicillin (LDP) induces sustained effects on body composition (e.g., tendency to obesity). Here, we re-examine a small portion of its original analyses, which address whether the LDP-affected microbial compositions are recovered after its cessation. For this, cecal microbiota were transferred from control-microbiota recipients (CR1) to seven germ-free mice (CR2) and LDP-microbiota recipients (PR1) to eight germ-free mice (PR2). Fecal specimens from these 15 recipient mice were collected 23 days after the transfer, and their DNA samples were analyzed by targeting the V4 region of the bacterial 16S rRNA gene. Using the QIIME pipeline [13] to quantify OTUs and construct a phylogenetic tree, 424 OTUs were observed, but after filtering OTUs with a proportion mean ≤10^−3^, 28 OTUs were analyzed.

We examined whether there is any disparity in microbial profiles between two groups (CR2 and PR2). No covariate adjustment was made assuming that other potential confounding factors were already well controlled in the randomized experimental design.

**Fig. 3:**
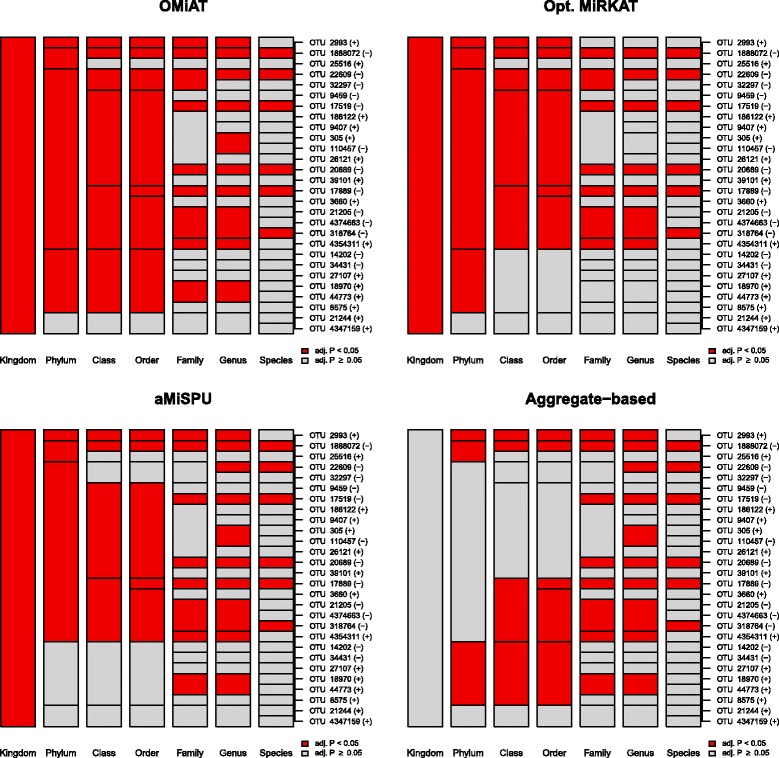
A hierarchical visualization which summarizes discovered (colored in *red*)/undiscovered (colored in *gray*) microbial taxa across different taxonomic ranks for the sustained effects of LDP on microbial profile using the group analytic methods, OMiAT, Optimal MiRKAT, aMiSPU, and the aggregate-based method

To summarize the results [Fig. 3, Additional file 7: Table S2], while many upper-level taxa were discovered consistently by the three methods, OMiAT, Optimal MiRKAT, and aMiSPU, the aggregate-based method discovered apparently less. Since many of the OTUs trend in opposite directions, the weakness of the aggregate-based method likely originates from the violation of its assumption of same effect directions of all associated OTUs. OMiAT discovered the greatest number of taxa, which is consistent with our simulations. The *P* values for testing the entire microbial community level were estimated as <0.001 for OMiAT, <0.001 for Optimal MiRKAT, <0.001 for aMiSPU, and 0.518 for the aggregate-based method.

#### Effects on intestinal microbiota by the early-life factor, vaginal or cesarean birth [7]

The early childhood antibiotics and the microbiome (ECAM) project is a longitudinal microbiome profiling study to examine the hypotheses that early life factors, such as delivery mode (e.g., vaginal or cesarean birth), infant nutrition (breast milk or formula predominance), and antibiotic usage, influence microbial community development, resulting in sustained states. Among 32 infants studied, 21 and 11 were delivered by vaginal and cesarean delivery, respectively. None had received antibiotics, and two covariate adjustments, predominant diet and sex, were included in our analyses.

The fecal samples from these infants were also assessed for the bacterial 16S rRNA V4 region, and OTUs were determined, and a phylogenetic tree was constructed [13]. There were 2261 OTUs in the original, but after filtering with a proportion mean ≤10^−3^, 71 OTUs were analyzed.

**Fig. 4:**
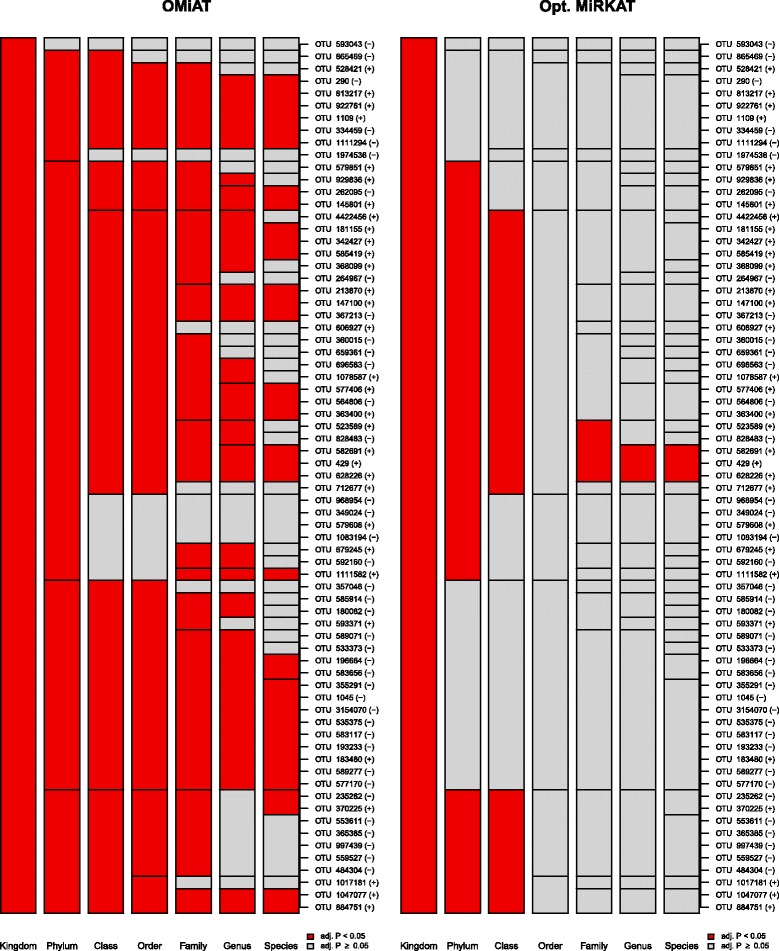
A hierarchical visualization which summarizes discovered (colored in *red*)/undiscovered (colored in *gray*) microbial taxa across different taxonomic ranks for the effects of delivery method on microbial profile using the group analytic methods, OMiAT and Optimal MiRKAT

**Fig. 5:**
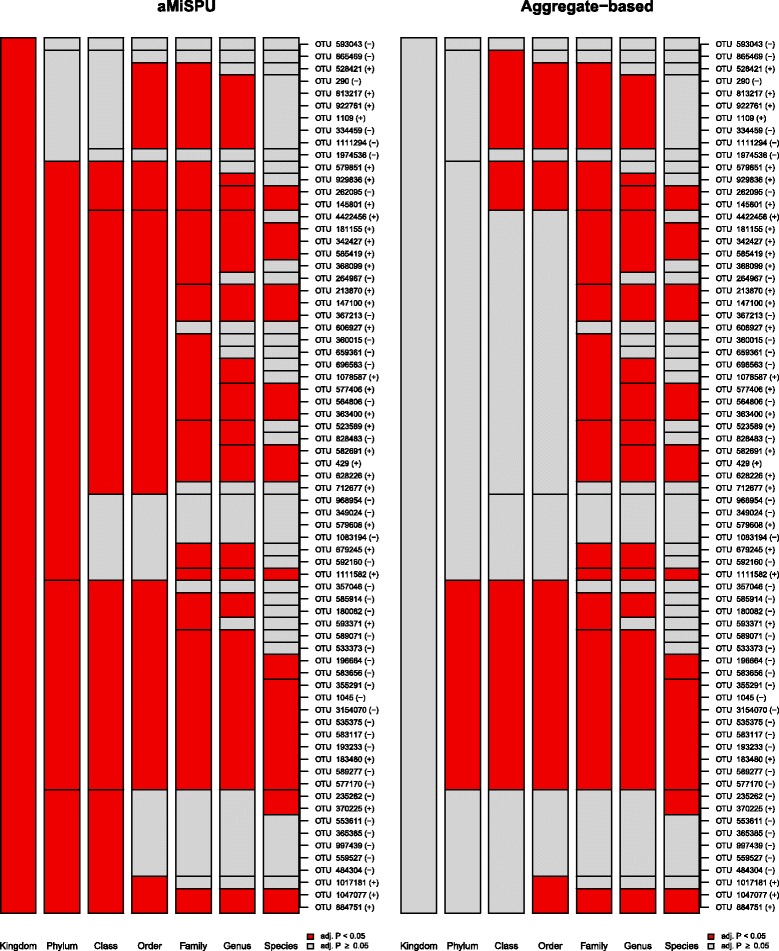
A hierarchical visualization which summarizes discovered (colored in *red*)/undiscovered (colored in *gray*) microbial taxa across different taxonomic ranks for the effects of delivery method on microbial profile using the group analytic methods, aMiSPU, and the aggregate-based method

We found that the aggregate-based method discovered apparently fewer microbial taxa than the other methods, since many of the OTUs had opposite effect directions [Figs. 4 and 5, Additional file 8: Table S3]. While many microbial taxa were consistently discovered by OMiAT and aMiSPU, many taxa do not overlap with Optimal MiRKAT [Figs. 4 and 5, Additional file 8: Table S3]. Some of the discovery statuses for the use of Optimal MiRKAT were also highly irregular by different specifications of the number of permutations since their *P* values were too close to 0.05. Here, again, OMiAT discovered the greatest number of taxa. The *P* values for testing the entire microbial community level were estimated as 0.005 for OMiAT, <0.001 for Optimal MiRKAT, 0.023 for aMiSPU, and 0.495 for the aggregate-based method.

## Discussion

The computational performance of MiCAM still needs to be improved. Although we could obtain stable outcomes efficiently for our real data analyses by the use of combined permutation-based algorithm, analyzing a big data may pose huge computational challenges in practice. To illustrate, as the number of microbial taxa increases (e.g., using a less stringent filtering rule), its computational burden can increase. MiCAM is written in R to facilitate the use of existing R functions, but in case of such huge computation, the use of a lower-level language can be required.

We have described the use of different group analytic methods and the MiCAM framework focusing on microbiota profiles via target sequencing for the 16S rRNA gene [10]. However, as long as a data includes OTU abundance and a phylogenetic tree in groups of interest, similar approaches can apply. Therefore, the extension to the shotgun metagenomic data for the whole microbial genomes [11, 46] is also highly feasible.

Although MiRKAT and SPU cover a wide range of association patterns, the candidate tests in the search space of OMiAT in Eq. 9 are not limited to those two sets of tests. If one finds other tests which suit other association patterns which are not covered by MiRKAT and SPU tests, one can include them into the search space to yield extra power.

As an extension, OMiAT can also be implemented into a hierarchical multiple testing scheme [47] to identify which microbes are associated with the phenotype of interest in the lowest taxonomic rank. By utilizing the taxonomic tree structure, one can test the lower-level lineages only when their upper-level taxon is significant. In this way, the number of tests can be reduced and smaller penalty due to multiple testing correction is needed.

## Conclusions

In this paper, we investigated two existing methods, MiRKAT and MiSPU, and a new method, OMiAT, that can be used as a counterpart to aggregate-based methods [21–24] in microbiome-based association studies. Due to the lack of knowledge about true underlying association patterns of numerous OTUs, the data-driven approaches (OMiAT, Optimal MiRKAT, and aMiSPU) are highly attractive in practice. We confirmed that they are all statistically valid approaches with well-controlled type I error rates. Among those, we observed that our proposed method, OMiAT, is most robust and powerful through extensive simulations and real data analyses. The high performance of OMiAT comes from its high adaptivity to suit two unique features of microbiome data, the high imbalance in microbial abundance and phylogenetic information.

The newly proposed microbial taxa discovery framework, MiCAM, organizes different configurations to test microbial taxa through a breadth of taxonomic ranks, and it is especially efficient for the assessment of upper-level taxa by integrating OMiAT as a group analytic method. Of importance is that MiCAM produces statistically significantly associated microbial taxa with a well-defined false discovery rate criterion. Its hierarchical visualization also helps rapidly overview multiple discovery statuses. Consequently, we can obtain a hierarchical association map for numerous microbial taxa, and this can also be used as a guideline for further investigation on the roles of discovered microbial taxa in human health and disease.

## Additional files

Additional file 1: Table S1. The permutation-based method to estimate *P* values for the test statistics, T_SPU(γ)_, T_aSPU_, Q_MiRKAT(k)_, Q_OMiRKAT_, and M_OMiAT_ [25, 31, 37]. (DOCX 23 kb)

Additional file 2: Figure S1. Type I error rate estimates for both linear and logistic models and for using the covariate, X_2_, as either correlated or independent with OTUs. (PDF 7 kb)

Additional file 3: Figure S2. Power estimates for the linear model using the covariate, X_2_, as independent with OTUs. (PDF 14 kb)

Additional file 4: Figure S3. Power estimates for the linear model using the covariate, X_2_, as correlated with OTUs. (PDF 14 kb)

Additional file 5: Figure S4. Power estimates for the logistic model using the covariate, X_2_, as independent with OTUs. (PDF 15 kb)

Additional file 6: Figure S5. Power estimates for the logistic model using the covariate, X_2_, as correlated with OTUs. (PDF 15 kb)

Additional file 7: Table S2. The names of the discovered microbial taxa using four methods to examine the sustained effects of LDP on microbial profiles. Discovered taxa without a name are excluded. (DOCX 15 kb)

Additional file 8: Table S3. The names of the discovered microbial taxa using four methods to examine the effects of birth mode on microbial profile. Discovered taxa without a name are excluded. (DOCX 14 kb)

## Abbreviations

ECAM: Early childhood antibiotics and the microbiome
LDP: Low-dose penicillin
LEfSe: Linear discriminant analysis effect size
MiCAM: Microbiome comprehensive association mapping
MiRKAT: Microbiome regression-based kernel association test
MiSPU: Microbiome-based sum of powered score tests (aMiSPU: adaptive MiSPU)
OMiAT: Optimal microbiome-based association test
OTU: Operational taxonomic unit
PAM: Partitioning-around-medoids
SD: Standard deviation
SPU: The sum of score powered tests (aSPU: adaptive SPU)
UniFrac: Unique fraction
