# Supplementary material for: A powerful microbiome-based association test and a microbial taxa discovery framework for comprehensive association mapping
Source: Microbiome. 2017 Apr 24;5:45. doi: 10.1186/s40168-017-0262-x (PMC5402681; doi:10.1186/s40168-017-0262-x)
Supplement: Supplementary file 1 — The permutation-based method to estimate P values for the test statistics, TSPU(γ), TaSPU, QMiRKAT(k), QOMiRKAT, and MOMiAT [25, 31, 37]. (DOCX 23 kb) [file 40168_2017_262_MOESM1_ESM.docx]

| 1) Two common steps for fitting a null model and permuting a vector of residuals:   - Step 1: Fit a null model (i.e., regress Y on the covariates) to obtain predicted values $\hat{\mu}_{i,0}$ and residuals$R_{i}=Y_{i}$ - $\hat{\mu}_{i,0}$ for i = 1, ..., n. - Step 2: Permute a vector of residuals R = ($R_{1}$, ..., $R_{n}$)' to obtain a permuted vector $R^{(b)}$, where b is an index (b ∈ {1, ..., B}) to identify each vector of residuals.   2) Calculate the P-value for $T_{SPU(\gamma)}$ denoted as $P_{SPU(\gamma)}$ [31]:   - i) Calculate $T_{SPU(\gamma)}$ as the summation of p elements in ${\{\sum_{i=1}^{n} {(Z}_{\mathrm{ij}}R_{i})}^{\gamma}$, j = 1, ..., p} (Equation 4). - ii) Calculate the null test statistic, $T_{SPU(\gamma)}^{(b)}$, based on each permuted residual vector and p predictors as the summation of p elements in ${\{\sum_{i=1}^{n} {(Z}_{\mathrm{ij}}R_{i}^{(b)})}^{\gamma}$, j = 1, ..., p} (Equation 4). - Repeat the above i) and ii) procedures for b = 1,..., B, and calculate the P-value, $P_{SPU(\gamma)}$, as $\sum_{b=1}^{B} [I({\vert T}_{SPU(\gamma)}^{(b)}$\| ≥ \|$T_{SPU(\gamma)}$\|)]/B, where I(·) is an indicator function.   3) Calculate the P-value for $T_{\mathrm{aSPU}}$, denoted as $P_{\mathrm{aSPU}}$ [31]:   - After calculating P-values, $P_{SPU(\gamma)}$, for each γ value following the procedures in 2), we can obtain $T_{\mathrm{aSPU}}$ as $T_{\mathrm{aSPU}}$ = $\min_{\gamma\epsilonГ} P_{SPU(\gamma)}$ (Equation 5). Then, using the same permuted vectors above, we can obtain the null test statistic, $T_{\mathrm{aSPU}}^{(b)}$, as $T_{\mathrm{aSPU}}^{(b)}$ = $\min_{\gamma\epsilonГ} \{\sum_{b1\neq b} [I(T_{\mathrm{SPU}\left( \gamma\right)}^{\left( b1 \right)}\geq T_{SPU(\gamma)}^{(b)}$) + 1]/(B-1)}, where b1 is an index (b1 ∈ {1, ..., B}) to identify each set of the residuals. Finally, we can obtain the P-value for $T_{\mathrm{aSPU}}$, $P_{\mathrm{aSPU}}$, as $P_{\mathrm{aSPU}}$ = $\sum_{b=1}^{B} [I(T_{\mathrm{aSPU}}^{(b)}$ ≤ $T_{\mathrm{aSPU}}$)]/B.   4) Calculate the P-value for $Q_{MiRKAT(k)}$ denoted as $P_{MiRKAT(k)}$ [25, 31]:   - i) Calculate $Q_{MiRKAT(k)}$ as$\frac{1}{2\Phi}$ R'K_(k)_R (Equation 6, 7). - ii) Calculate the null test statistic, $Q_{MiRKAT(k)}^{(b)}$, based on each permuted residual vector as $\frac{1}{2\Phi}$ ${(R}^{(b)})'$K_(k)_$R^{(b)}$ (Equation 6, 7). - Repeat the above steps for b = 1,..., B, and calculate the P-value, $P_{MiRKAT(k)}$, as $\sum_{b=1}^{B} [I({\vert Q}_{MiRKAT(k)}^{(b)}$\| ≥ \|$Q_{MiRKAT(k)}$\|)]/B, where I(·) is an indicator function.   5) Calculate the P-value for $Q_{\mathrm{OMiRKAT}}$, denoted as $P_{\mathrm{OMiRKAT}}$ [25, 31]:   - After calculating P-values, $P_{MiRKAT(k)}$, for each k kernel following the procedures in 4), we can obtain $Q_{\mathrm{OMiRKAT}}$ as $Q_{\mathrm{OMiRKAT}}$ = $\min_{k\epsilon\{1, \ldots, l\}} P_{MiRKAT(k)}$ (Equation 8). Then, using the same permuted vectors above, we can obtain the null test statistic, $Q_{\mathrm{OMiRKAT}}^{(b)}$, as $Q_{\mathrm{OMiRKAT}}^{(b)}$ = $\min_{k\epsilon\{1, \ldots, l\}} \{\sum_{b1\neq b} [I(Q_{\mathrm{MiRKAT}\left( k \right)}^{\left( b1 \right)}\geq Q_{MiRKAT(k)}^{(b)}$)]/(B-1)}, where b1 is an index (b1 ∈ {1, ..., B}) to identify each vector of the residuals. Finally, we can obtain the P-value for $Q_{\mathrm{OMiRKAT}}$, $P_{\mathrm{OMiRKAT}}$, as $P_{\mathrm{OMiRKAT}}$ = $\sum_{b=1}^{B} [I(Q_{\mathrm{OMiRKAT}}^{(b)}$ ≤ $Q_{\mathrm{OMiRKAT}}$)]/B.   6) Calculate the P-value for $M_{\mathrm{OMiAT}}$, denoted as $P_{\mathrm{OMiAT}}$:   - Following the procedures, 1)-5), we can obtain $M_{\mathrm{OMiAT}}$ as $M_{\mathrm{OMiAT}}$ = min($T_{\mathrm{aSPU}}$, $Q_{\mathrm{OMiRKAT}}$). Then, we take $M_{\mathrm{OMiAT}}^{(b)}$ as $M_{\mathrm{OMiAT}}^{(b)}$ = min($T_{\mathrm{aSPU}}^{(b)}$, $Q_{\mathrm{OMiRKAT}}^{(b)}$). Finally, we can obtain the P-value for $M_{\mathrm{OMiAT}}$, $P_{\mathrm{OMiAT}}$ as $P_{\mathrm{OMiAT}}$ = $\sum_{b=1}^{B} [I(M_{\mathrm{OMiAT}}^{(b)}$ ≤ $M_{\mathrm{OMiAT}}$)]/B. |
| --- |
