# Supplementary material for: A powerful microbiome-based association test and a microbial taxa discovery framework for comprehensive association mapping
Source: Microbiome. 2017 Apr 24;5:45. doi: 10.1186/s40168-017-0262-x (PMC5402681; doi:10.1186/s40168-017-0262-x)
Supplement: Supplementary file 2 — Type I error rate estimates for both linear and logistic models and for using the covariate, X2, as either correlated or independent with OTUs. (PDF 7 kb) [file 40168_2017_262_MOESM2_ESM.pdf]

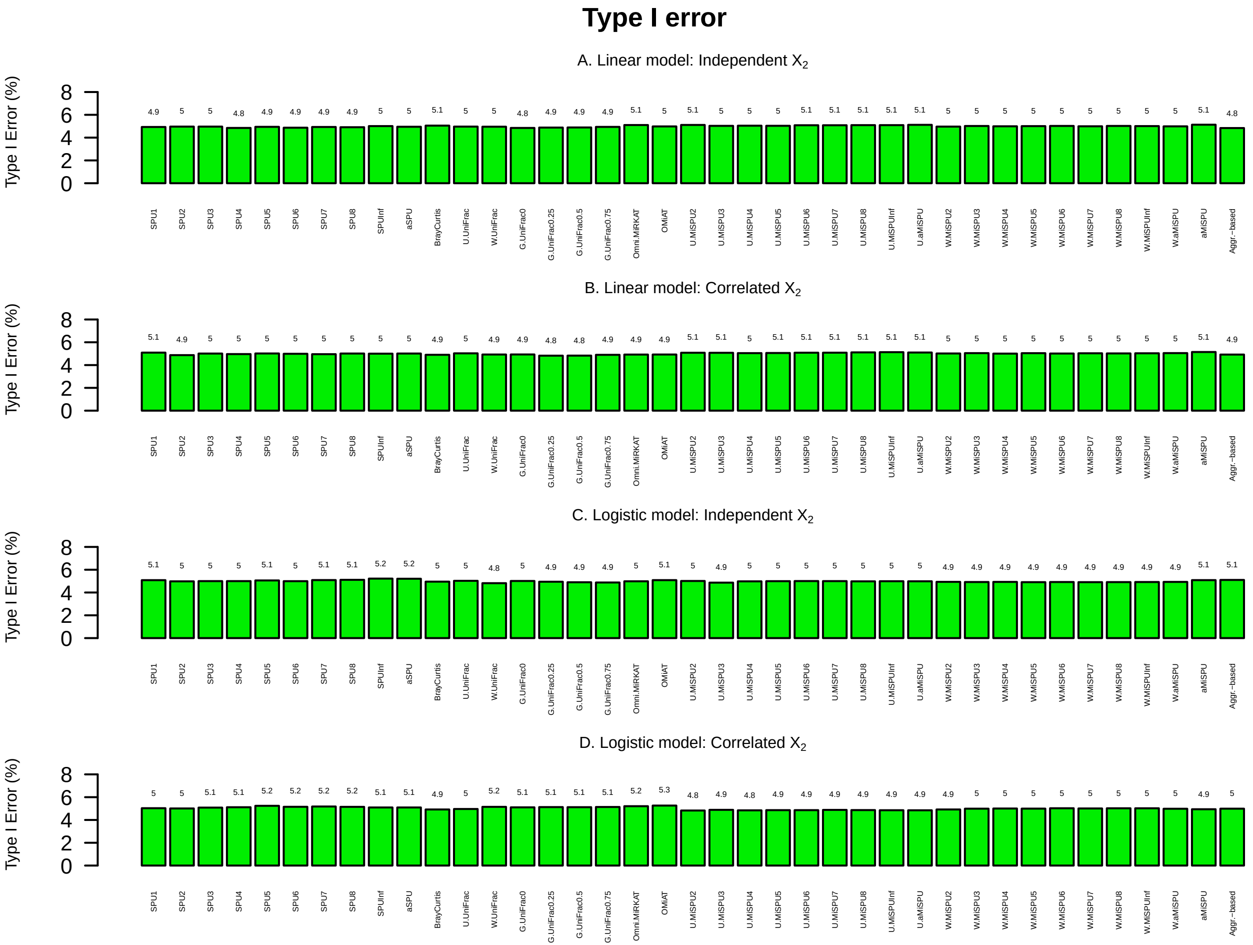
Type I error
A. Linear model: Independent X_2
Type I Error (%)
0 2 4 6 8
4.9 5 5 4.8 4.9 4.9 4.9 4.9 5 5 5.1 5 5 4.8 4.9 4.9 4.9 5.1 5 5.1 5 5 5 5.1 5.1 5.1 5.1 5.1 5 5 5 5 5 5 5 5 5 5.1 4.8
SPU1 SPU2 SPU3 SPU4 SPU5 SPU6 SPU7 SPU8 SPUInf aSPU BrayCurtis U.UniFrac W.UniFrac G.UniFrac0 G.UniFrac0.25 G.UniFrac0.5 G.UniFrac0.75 Omni.MiRKAT OMiAT U.MiSPU2 U.MiSPU3 U.MiSPU4 U.MiSPU5 U.MiSPU6 U.MiSPU7 U.MiSPU8 U.MiSPUInf U.aMiSPU W.MiSPU2 W.MiSPU3 W.MiSPU4 W.MiSPU5 W.MiSPU6 W.MiSPU7 W.MiSPU8 W.MiSPUInf W.aMiSPU aMiSPU Aggr.−based
B. Linear model: Correlated X_2
Type I Error (%)
0 2 4 6 8
5.1 4.9 5 5 5 5 5 5 5 5 4.9 5 4.9 4.9 4.8 4.8 4.9 4.9 4.9 5.1 5.1 5 5.1 5.1 5.1 5.1 5.1 5.1 5 5 5 5 5 5 5 5 5 5.1 4.9
SPU1 SPU2 SPU3 SPU4 SPU5 SPU6 SPU7 SPU8 SPUInf aSPU BrayCurtis U.UniFrac W.UniFrac G.UniFrac0 G.UniFrac0.25 G.UniFrac0.5 G.UniFrac0.75 Omni.MiRKAT OMiAT U.MiSPU2 U.MiSPU3 U.MiSPU4 U.MiSPU5 U.MiSPU6 U.MiSPU7 U.MiSPU8 U.MiSPUInf U.aMiSPU W.MiSPU2 W.MiSPU3 W.MiSPU4 W.MiSPU5 W.MiSPU6 W.MiSPU7 W.MiSPU8 W.MiSPUInf W.aMiSPU aMiSPU Aggr.−based
C. Logistic model: Independent X_2
Type I Error (%)
0 2 4 6 8
5.1 5 5 5 5.1 5 5.1 5.1 5.2 5.2 5 5 4.8 5 4.9 4.9 4.9 5 5.1 5 4.9 5 5 5 5 5 5 5 4.9 4.9 4.9 4.9 4.9 4.9 4.9 4.9 4.9 5.1 5.1
SPU1 SPU2 SPU3 SPU4 SPU5 SPU6 SPU7 SPU8 SPUInf aSPU BrayCurtis U.UniFrac W.UniFrac G.UniFrac0 G.UniFrac0.25 G.UniFrac0.5 G.UniFrac0.75 Omni.MiRKAT OMiAT U.MiSPU2 U.MiSPU3 U.MiSPU4 U.MiSPU5 U.MiSPU6 U.MiSPU7 U.MiSPU8 U.MiSPUInf U.aMiSPU W.MiSPU2 W.MiSPU3 W.MiSPU4 W.MiSPU5 W.MiSPU6 W.MiSPU7 W.MiSPU8 W.MiSPUInf W.aMiSPU aMiSPU Aggr.−based
D. Logistic model: Correlated X_2
Type I Error (%)
0 2 4 6 8
5 5 5.1 5.1 5.2 5.2 5.2 5.2 5.1 5.1 4.9 5 5.2 5.1 5.1 5.1 5.1 5.2 5.3 4.8 4.9 4.8 4.9 4.9 4.9 4.9 4.9 4.9 4.9 5 5 5 5 5 5 5 5 4.9 5
SPU1 SPU2 SPU3 SPU4 SPU5 SPU6 SPU7 SPU8 SPUInf aSPU BrayCurtis U.UniFrac W.UniFrac G.UniFrac0 G.UniFrac0.25 G.UniFrac0.5 G.UniFrac0.75 Omni.MiRKAT OMiAT U.MiSPU2 U.MiSPU3 U.MiSPU4 U.MiSPU5 U.MiSPU6 U.MiSPU7 U.MiSPU8 U.MiSPUInf U.aMiSPU W.MiSPU2 W.MiSPU3 W.MiSPU4 W.MiSPU5 W.MiSPU6 W.MiSPU7 W.MiSPU8 W.MiSPUInf W.aMiSPU aMiSPU Aggr.−based
