# Supplementary material for: A powerful microbiome-based association test and a microbial taxa discovery framework for comprehensive association mapping
Source: Microbiome. 2017 Apr 24;5:45. doi: 10.1186/s40168-017-0262-x (PMC5402681; doi:10.1186/s40168-017-0262-x)

Linear model: Independent X_2

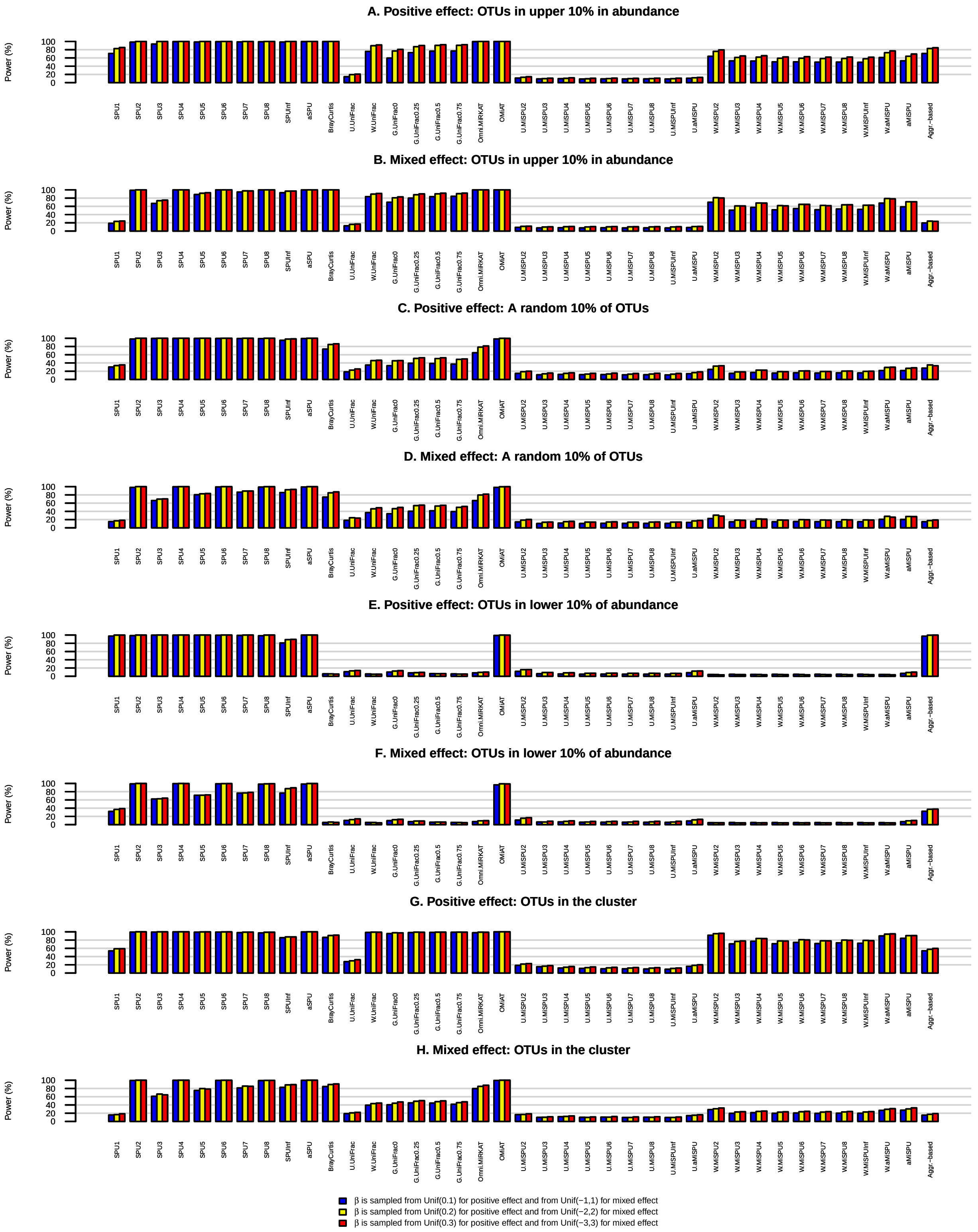

Supplement: Supplementary file 3 — Power estimates for the linear model using the covariate, X2, as independent with OTUs. (PDF 14 kb) [file 40168_2017_262_MOESM3_ESM.pdf]
