# Supplementary material for: A powerful microbiome-based association test and a microbial taxa discovery framework for comprehensive association mapping
Source: Microbiome. 2017 Apr 24;5:45. doi: 10.1186/s40168-017-0262-x (PMC5402681; doi:10.1186/s40168-017-0262-x)
Supplement: Supplementary file 4 — Power estimates for the linear model using the covariate, X2, as correlated with OTUs. (PDF 14 kb) [file 40168_2017_262_MOESM4_ESM.pdf]

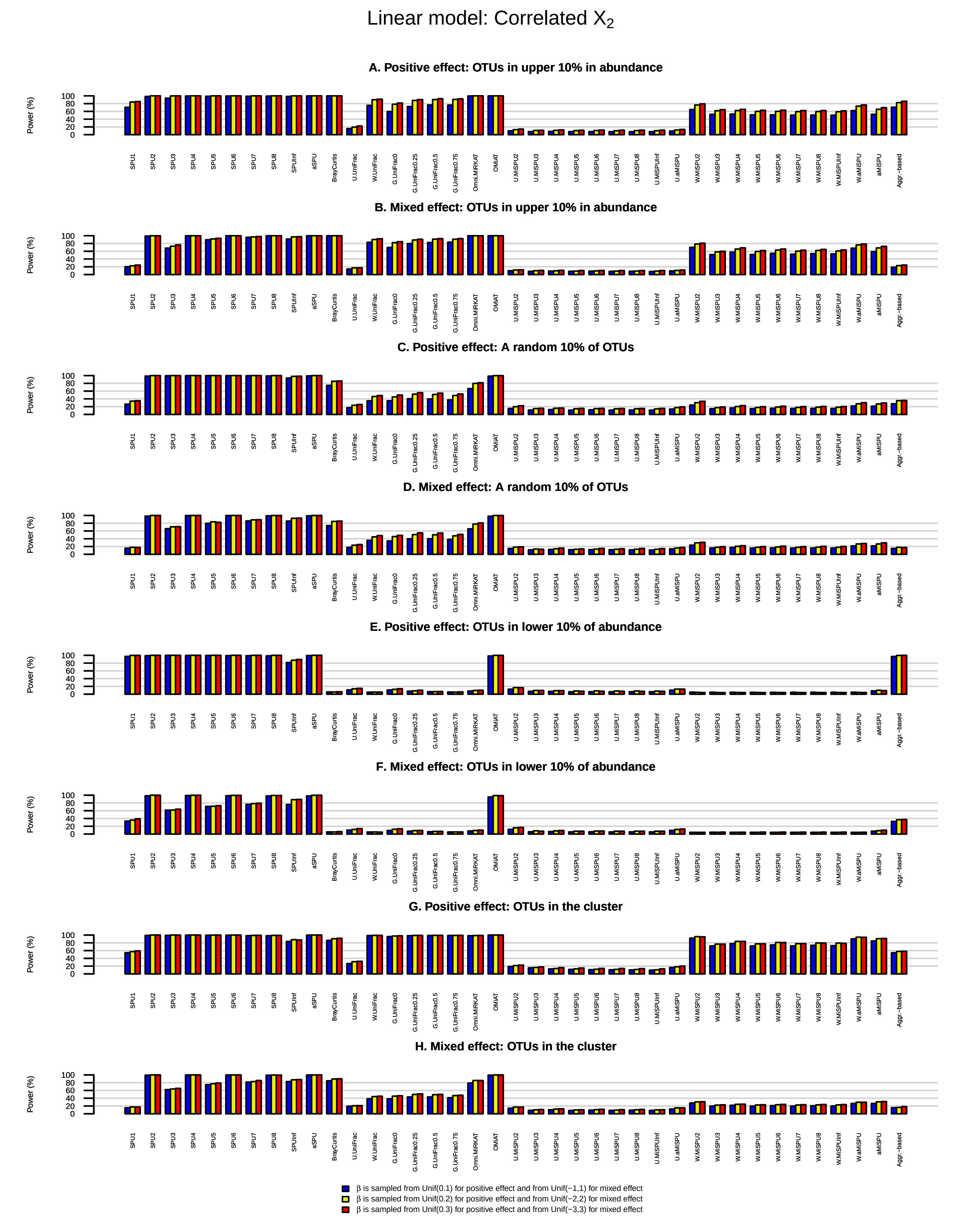
Linear model: Correlated X_2
A. Positive effect: OTUs in upper 10% in abundance
B. Mixed effect: OTUs in upper 10% in abundance
C. Positive effect: A random 10% of OTUs
D. Mixed effect: A random 10% of OTUs
E. Positive effect: OTUs in lower 10% of abundance
F. Mixed effect: OTUs in lower 10% of abundance
G. Positive effect: OTUs in the cluster
H. Mixed effect: OTUs in the cluster
Power (%)
SPU1
SPU2
SPU3
SPU4
SPU5
SPU6
SPU7
SPU8
SPUInf
aSPU
BrayCurtis
U.UniFrac
W.UniFrac
G.UniFrac0
G.UniFrac0.25
G.UniFrac0.5
G.UniFrac0.75
Omni.MiRKAT
OMiAT
U.MiSPU2
U.MiSPU3
U.MiSPU4
U.MiSPU5
U.MiSPU6
U.MiSPU7
U.MiSPU8
U.MiSPUInf
U.aMiSPU
W.MiSPU2
W.MiSPU3
W.MiSPU4
W.MiSPU5
W.MiSPU6
W.MiSPU7
W.MiSPU8
W.MiSPUInf
W.aMiSPU
aMiSPU
Aggr.−based
β is sampled from Unif(0.1) for positive effect and from Unif(−1,1) for mixed effect
β is sampled from Unif(0.2) for positive effect and from Unif(−2,2) for mixed effect
β is sampled from Unif(0.3) for positive effect and from Unif(−3,3) for mixed effect
