# Supplementary material for: A powerful microbiome-based association test and a microbial taxa discovery framework for comprehensive association mapping
Source: Microbiome. 2017 Apr 24;5:45. doi: 10.1186/s40168-017-0262-x (PMC5402681; doi:10.1186/s40168-017-0262-x)

# Logistic model: Correlated $X_2$

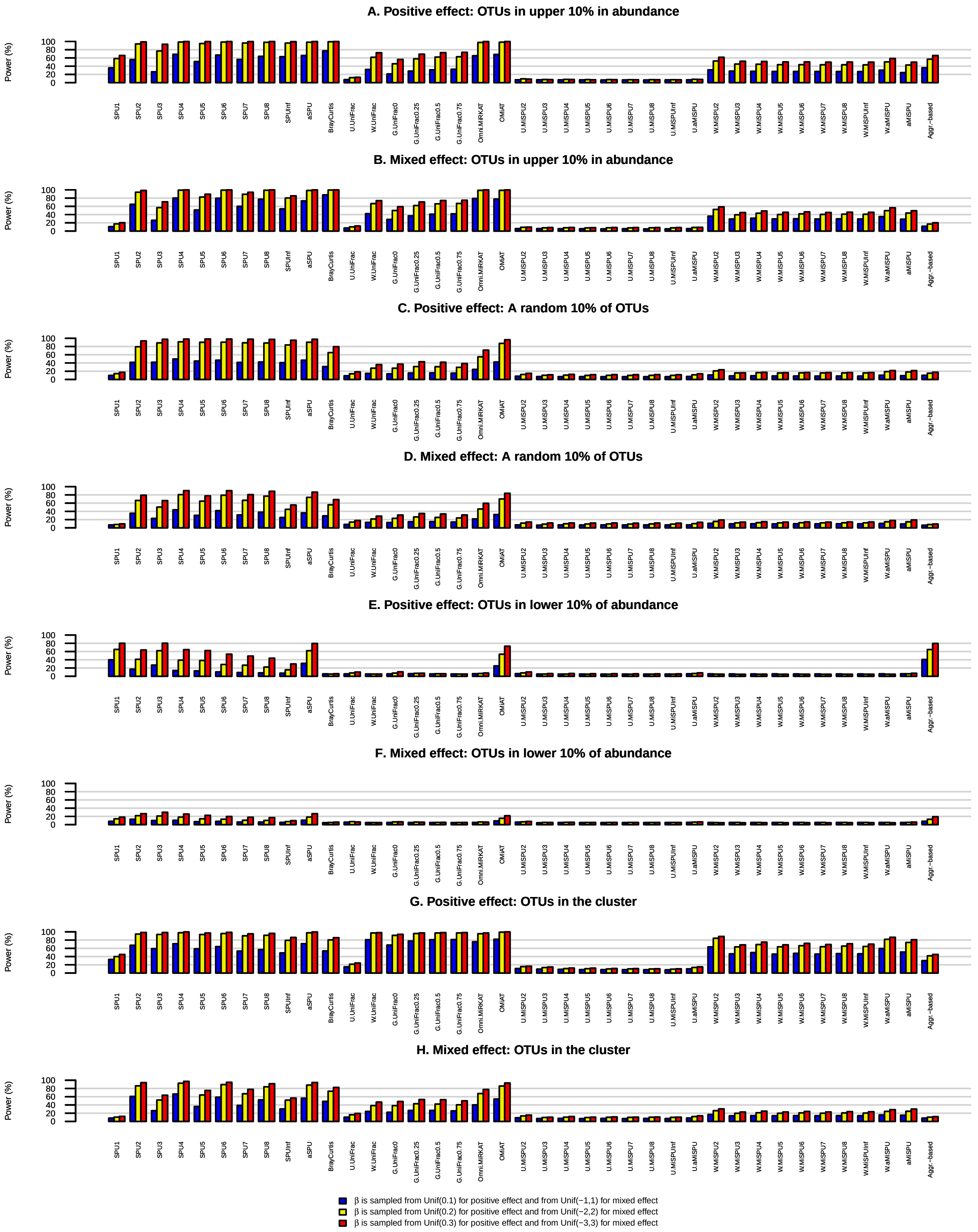

Supplement: Supplementary file 6 — Power estimates for the logistic model using the covariate, X2, as correlated with OTUs. (PDF 15 kb) [file 40168_2017_262_MOESM6_ESM.pdf]
