# Supplementary material for: A powerful microbiome-based association test and a microbial taxa discovery framework for comprehensive association mapping
Source: Microbiome. 2017 Apr 24;5:45. doi: 10.1186/s40168-017-0262-x (PMC5402681; doi:10.1186/s40168-017-0262-x)
Supplement: Supplementary file 7 — The names of the discovered microbial taxa using four methods to examine the sustained effects of LDP on microbial profiles. Discovered taxa without a name are excluded. (DOCX 15 kb) [file 40168_2017_262_MOESM7_ESM.docx]

|  | **OMiAT** | **Opt. MiRKAT** | **aMiSPU** | **Aggregate-based** |
| --- | --- | --- | --- | --- |
| **Kingdom** | *Bacteria* | *Bacteria* | *Bacteria* |  |
| **Phylum** | *Bacteroidetes*  *Firmicutes*  *Proteobacteria*  *Verrucomicrobia* | *Bacteroidetes*  *Firmicutes*  *Proteobacteria*  *Verrucomicrobia* | *Firmicutes*  *Proteobacteria*  *Verrucomicrobia* | *Bacteroidetes*  *Proteobacteria*  *Verrucomicrobia* |
| **Class** | *Bacteroidia*  *Bacilli*  *Clostridia*  *Erysipelotrichi*  *Gammaproteobacteria*  *Verrucomicrobiae* | *Bacilli*  *Clostridia*  *Erysipelotrichi*  *Gammaproteobacteria*  *Verrucomicrobiae* | *Bacilli*  *Clostridia*  *Gammaproteobacteria*  *Verrucomicrobiae* | *Bacteroidia*  *Bacilli*  *Gammaproteobacteria*  *Verrucomicrobiae* |
| **Order** | *Bacteroidales*  *Lactobacillales*  *Turicibacterales*  *Clostridiales*  *Erysipelotrichales*  *Enterobacteriales*  *Verrucomicrobiales* | *Lactobacillales*  *Turicibacterales*  *Clostridiales*  *Erysipelotrichales*  *Enterobacteriales*  *Verrucomicrobiales* | *Lactobacillales*  *Turicibacterales*  *Clostridiales*  *Enterobacteriales*  *Verrucomicrobiales* | *Bacteroidales*  *Lactobacillales*  *Turicibacterales*  *Enterobacteriales*  *Verrucomicrobiales* |
| **Family** | *Bacteroidaceae*  *Enterococcaceae*  *Lactobacillaceae*  *Turicibacteraceae*  *Clostridiaceae*  *Peptostreptococcaceae*  *Erysipelotrichaceae*  *Enterobacteriaceae*  *Verrucomicrobiaceae* | *Enterococcaceae*  *Lactobacillaceae*  *Turicibacteraceae*  *Clostridiaceae*  *Peptostreptococcaceae*  *Erysipelotrichaceae*  *Enterobacteriaceae*  *Verrucomicrobiaceae* | *Bacteroidaceae*  *Enterococcaceae*  *Lactobacillaceae*  *Turicibacteraceae*  *Clostridiaceae*  *Peptostreptococcaceae*  *Enterobacteriaceae*  *Verrucomicrobiaceae* | *Bacteroidaceae*  *Enterococcaceae*  *Lactobacillaceae*  *Turicibacteraceae*  *Clostridiaceae*  *Peptostreptococcaceae*  *Enterobacteriaceae*  *Verrucomicrobiaceae* |
| **Genus** | *Bacteroides*  *Enterococcus*  *Lactobacillus*  *Turicibacter*  *Blautia*  *Allobaculum*  *Klebsiella*  *Akkermansia* | *Enterococcus*  *Lactobacillus*  *Turicibacter*  *Allobaculum*  *Klebsiella* | *Bacteroides*  *Enterococcus*  *Lactobacillus*  *Turicibacter*  *Blautia*  *Allobaculum*  *Klebsiella*  *Akkermansia* | *Bacteroides*  *Enterococcus*  *Lactobacillus*  *Turicibacter*  *Blautia*  *Allobaculum*  *Klebsiella*  *Akkermansia* |
| **Species** |  |  |  |  |
