# Supplementary material for: A powerful microbiome-based association test and a microbial taxa discovery framework for comprehensive association mapping
Source: Microbiome. 2017 Apr 24;5:45. doi: 10.1186/s40168-017-0262-x (PMC5402681; doi:10.1186/s40168-017-0262-x)
Supplement: Supplementary file 8 — The names of the discovered microbial taxa using four methods to examine the effects of birth mode on microbial profile. Discovered taxa without a name are excluded. (DOCX 14 kb) [file 40168_2017_262_MOESM8_ESM.docx]

|  | **OMiAT** | **Opt. MiRKAT** | **aMiSPU** | **Aggregate-based** |
| --- | --- | --- | --- | --- |
| **Kingdom** | *Bacteria* | *Bacteria* | *Bacteria* |  |
| **Phylum** | *Actinobacteria*  *Bacteroidetes*  *Firmicutes*  *Proteobacteria* | *Actinobacteria*  *Firmicutes* | *Actinobacteria*  *Bacteroidetes*  *Firmicutes* | *Bacteroidetes* |
| **Class** | *Actinobacteria*  *Bacteroidia*  *Clostridia*  *Erysipelotrichi*  *Gammaproteobacteria* | *Actinobacteria*  *Clostridia* | *Actinobacteria*  *Bacteroidia*  *Clostridia*  *Erysipelotrichi* | *Bacteroidia*  *Erysipelotrichi*  *Gammaproteobacteria* |
| **Order** | *Actinomycetales*  *Bifidobacteriales*  *Bacteroidales*  *Clostridiales*  *Erysipelotrichales*  *Enterobacteriales* |  | *Actinomycetales*  *Bacteroidales*  *Clostridiales*  *Erysipelotrichales*  *Enterobacteriales* | *Actinomycetales*  *Bacteroidales*  *Erysipelotrichales*  *Enterobacteriales* |
| **Family** | *Actinomycetaceae*  *Bifidobacteriaceae*  *Bacteroidaceae*  *Porphyromonadaceae*  *Enterococcaceae*  *Lactobacillaceae*  *Clostridiaceae*  *Lachnospiraceae*  *Ruminococcaceae*  *Veillonellaceae*  *Erysipelotrichaceae*  *Enterobacteriaceae* | *Clostridiaceae* | *Actinomycetaceae*  *Bacteroidaceae*  *Porphyromonadaceae*  *Enterococcaceae*  *Lactobacillaceae*  *Clostridiaceae*  *Lachnospiraceae*  *Ruminococcaceae*  *Veillonellaceae*  *Erysipelotrichaceae*  *Enterobacteriaceae* | *Actinomycetaceae*  *Bacteroidaceae*  *Porphyromonadaceae*  *Enterococcaceae*  *Lactobacillaceae*  *Clostridiaceae*  *Lachnospiraceae*  *Ruminococcaceae*  *Veillonellaceae*  *Erysipelotrichaceae*  *Enterobacteriaceae* |
| **Genus** | *Actinomyces*  *Bacteroides*  *Parabacteroides*  *Enterococcus*  *Lactobacillus*  *Clostridium*  *Blautia*  *Veillonella*  *Coprobacillus* |  | *Actinomyces*  *Bacteroides*  *Parabacteroides*  *Enterococcus*  *Lactobacillus*  *Clostridium*  *Blautia*  *Veillonella*  *Coprobacillus* | *Actinomyces*  *Bacteroides*  *Parabacteroides*  *Enterococcus*  *Lactobacillus*  *Clostridium*  *Blautia*  *Veillonella*  *Coprobacillus* |
| **Species** | *adolescentis*  *fragilis*  *dispar* |  | *adolescentis*  *fragilis*  *dispar* | *adolescentis*  *fragilis*  *dispar* |
